# Live attenuated *Salmonella* displaying HIV-1 10E8 epitope on fimbriae: systemic and mucosal immune responses in BALB/c mice by mucosal administration

**DOI:** 10.1038/srep29556

**Published:** 2016-07-14

**Authors:** Qing-Hai Li, Gang Jin, Jia-Ye Wang, Hai-Ning Li, Huidi Liu, Xiao-Yun Chang, Fu-Xiang Wang, Shu-Lin Liu

**Affiliations:** 1 Systemomics Center, College of Pharmacy, and Genomics Research Center (State-Province Key Laboratories of Biomedicine-Pharmaceutics of China), Harbin Medical University, Harbin, China; 2 Department of Microbiology, Harbin Medical University, Harbin, China; 3 Department of Infectious Diseases, The Fourth Affiliated Hospital of Harbin Medical University, Harbin, China; 4 Department of Infectious Diseases, The First Affiliated Hospital, Harbin Medical University, Harbin, China; 5 Department of Microbiology, Immunology and Infectious Diseases, University of Calgary, Calgary, Canada

## Abstract

The HIV-1 membrane proximal external region (MPER) that is targeted by several broadly neutralizing antibodies (BNAbs) has been considered a potential immunogen for vaccine development. However, to date the immunogenicity of these BNAb epitopes has not been made sufficiently adequate. In the present work, we used live attenuated *Salmonella* as a platform to present the HIV-1 MPER 10E8 epitope in the fimbriae. The insertion of the 10E8 epitope into the fimbriae had no significant influence on the expression and the absorption capacity of bacterial fimbriae, nor on the virulence and invasiveness of the attenuated *Salmonella*. After oral administration of the vaccine construct to mice followed by 10E8 epitope peptide boost, specific antibody responses in serum and mucosa as well as memory lymphocytes in spleen and plasma cells in bone marrow were induced. We also found that the live attenuated *Salmonella* vector directed the immunity toward Th1 bias, induced Th1 and Th2 cytokine responses and stimulated significant B cell differentiation into GC B, memory B and plasma cells. Therefore, we propose that the live attenuated *Salmonella* constitutively expressing HIV-1 BNAb epitopes on the fimbriae will be an effective approach to improving immune microenvironment and enhancing the immunogenicity of HIV-1 epitope vaccines.

Developing effective vaccines against human immunodeficiency virus type 1 (HIV-1) still faces considerable challenges three decades after the discovery of the virus^1^,^2^. Enormous and persistent efforts have been made to generate many vaccine candidates, including the vaccine in trial RV144 that showed modest protection^3^,^4^, but so far none has shown the effects useful for human vaccination. Recent studies on HIV-1 pathogenesis and vaccinology have given rise to a general consensus for vaccine design that an ideal HIV-1 vaccine paradigm should simulate the natural route of viral infection^5^,^6^,^7^. Since the primary site of HIV-1 infection is the mucosal surface^8^,^9^, the elicitation of protective antibody (neutralizing or non-neutralizing antibody) responses not only in systemic but also in mucosal compartments should be taken into consideration for HIV-1 vaccine design.

Most naturally infected individuals develop strain-specific antibodies against HIV-1; of interest, approximately 5–25% of HIV-1 infected individuals may develop broadly neutralizing antibodies (BNAbs), which is the primary goal for prophylactic HIV-1 vaccines^10^,^11^,^12^. Since 2009, a growing number of BNAbs have been reported, with most showing wide breadth and strong potency^13^,^14^. The targets of such antibodies on the HIV-1 envelope glycoprotein (Env) can be grouped into three parts: gp120, gp41 and the interface region of gp120-gp41^13^,^15^. Thus, epitopes identified in these regions have been chosen as immunogens for HIV-1 vaccine designs. Among them, the membrane proximal external region (MPER) of gp41 subunit has been known as one of the most highly conserved sequences, contributing to the HIV-1 viral fusion and infectivity^16^, and the epitopes of all three well-characterized BNAbs, 2F5, 4E10, and 10E8, are in MPER. Unfortunately, although these epitopes seem to be ideal for vaccine development due to their particularly suitable features such as being highly conserved, linear and easily accessible to immune molecules, many MPER-based vaccine strategies incorporating these epitopes in different conformations have resulted in merely weak or no epitope-specific neutralizing antibody responses *in vivo*. The main problem has been in the presentation manner of the epitopes to maximally preserve their high immunogenicity and some other natural features. Diverse approaches have been tried for this, including the scaffold protein method^17^,^18^,^19^, nanoparticle techniques^20^, and virus-like particle strategies^21^. However, all these approaches have only slightly improved the immunogenicity of the MPRE epitopes, mainly because of the very limited numbers of antigenic epitopes to be displayed on the surface and short retention time of the vaccine vectors, which leads to inadequate stimulation to the immune system.

Recombinant bacterial vectors such as live attenuated *Salmonella*^22^,^23^,^24^ seem to be a solution, due to their ability to invade and colonize tissues after mucosal delivery^25^,^26^. Upon infection and invasion of the intestinal mucosa, the *Salmonella* bacteria are taken up by macrophages or other antigen-presenting cells and will then grow inside these cells^27^. Such features can allow for persistent exposure of vaccinated individuals to heterologous immunogens. Additionally, *Salmonella* infection will at the same time stimulate innate immunity, such as the activation of macrophages and the recruitment of other immune cells. In light of such advantages, *Salmonella* may be used as an ideal vector for the development of HIV-1 vaccines.

The MPER epitopes are hydrophobic components of the HIV-1 envelop glycoprotein, so the epitope peptide should be presented on the outer membrane component of the bacterial vectors in the vaccine to be developed. The *Salmonella* thin aggregative fimbriae can precisely satisfy this requirement compared to other strategies that have been described to date^24^,^28^,^29^,^30^. In the present study, we constructed live attenuated *Salmonella* that constitutively express the HIV-1 10E8 epitope on the thin aggregative fimbriae. After confirming the successful construction, we immunized mice by using different vaccination regimens and found that the recombinant vaccine *Salmonella* strain induced high levels of humoral and mucosal epitope-specific Ab responses and high frequency of B cells and plasma cells secreting antibodies specific to HIV-1.

## Results

### Characterization of recombinant attenuated *Salmonella* expressing HIV-1 10E8 epitope

**Figure 1:**
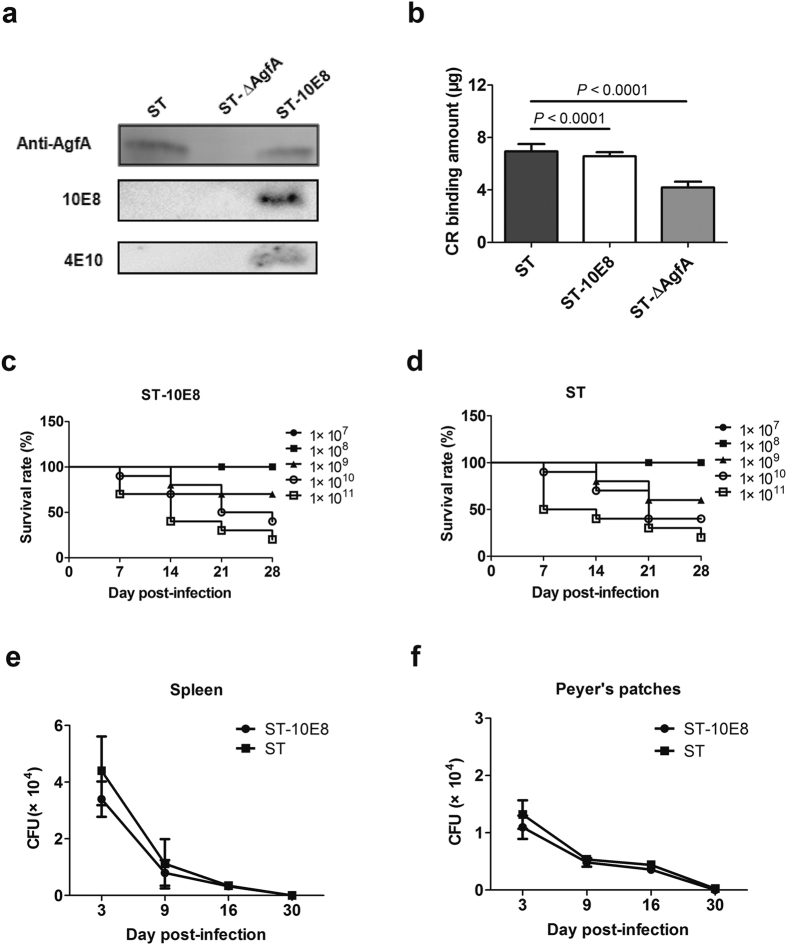
Characterization of live attenuated recombinant *Salmonella* expressing HIV-1 10E8 epitope on fimbriae. (**a**) Expression of chimeric AgfA::10E8 protein in ST-10E8 strain detected by western blot analysis using anti-AgfA polyclonal sera, and mAbs 10E8 and 4E10. (**b**) Congo red tests on ST-10E8, ST and AgfA-deficient strain (ST-∆AgfA). (**c**,**d**) Survival rates of mice (10 per group) infected with ST-10E8 or ST strain from 10^7^ to 10^11^ CFU. (**e**,**f**) Colonization of ST-10E8 or ST strain in spleen and Peyer’s patches (12 mice per group). Data shown as mean ± SD of 5 mice in each group.

We employed the scarless and efficient gene replacement system, using the temperature-sensitive plasmid pHSG415 as a carrier of foreign genes, to integrate the DNA fragment encoding the HIV-1 10E8 linear epitope into the fimbrin gene *agfA* of attenuated *Salmonella typhimurium*, ST-14028 3b *aroA*^−^ (referred to as ST hereafter). DNA sequencing confirmed that recombinant *S. typhimurium* (ST-10E8) contained chimeric *agfA::10E8* gene. To verify the expression of the chimeric fimbrial protein, we detected the AgfA and 10E8 by western blotting analysis and found that both AgfA protein and 10E8 epitope were present on the recombinant ST-10E8 strain (Fig. 1a).

### ST-10E8 showed comparable Congo red binding activity to ST

To assess the intactness of the aggressive fimbriae that have the 10E8 epitope inserted, we conducted CR binding assay by using the hydrophobic Congo red dye (CR), the binding activity of which to *Salmonella* is commensurate with the amounts of aggressive fimbriae, and found that the CR bound by ST-10E8 was similar to that by ST. As expected, significantly less CR was bound by the AgfA-deficient ST (ST-ΔAgfA) (Fig. 1b).

### ST-10E8 showed comparable virulence and invasion ability to those of ST

Considering that fimbriae contribute to the primary tissue attachment process of *Salmonella*, we wanted to know whether the modified fimbriae could influence the virulence such as invasiveness of ST. We infected the BALB/c mice (8–10 week old) orally with ST or ST-10E8 at doses from 10^7^ to 10^11^ colony forming units (CFU) and found that these two groups had comparable survival rates at the same dose over a 4-week period (Fig. 1c,d). The 50% lethal doses (LD50) of ST and ST-10E8 in BALB/c mice were determined as 5.0 × 10^9^ CFU and 6.3 × 10^9^ CFU, respectively.

After infection with 1 × 10^8^ CFU of ST-10E8 or ST, similar bacterial colonization status of these STs in lymphoid tissues was observed. Bacterial colonization in spleens was much heavier than that in Peyer’s patches at the same time points. The bacterial loads of ST-10E8 were rapidly decreased from 3.4 × 10^4^ CFU in spleen and 1.1 × 10^4^ CFU in Peyer’s patches at day 3 to 8.0×10^3^ CFU and 4.8 × 10^3^ CFU at day 9, respectively. Since then, bacterial loads were maintained at a low level and most of the bacteria were cleared out at day 30 after infection (Fig. 1e,f).

Together, these results indicated that the attenuated *Salmonella* ST-10E8 could display the HIV-1 10E8 epitope on functional aggressive fimbrial AgfA protein. Of special importance, the insertion of the foreign 10E8 epitope into the fimbriae had no significant influence on the expression of AgfA protein, the absorption capacity of bacterial fimbriae, or virulence such as the invasiveness of the attenuated *Salmonella* ST.

### Oral immunization with ST-10E8 induced 10E8 epitope specific systemic and mucosal immune memory

**Figure 2:**
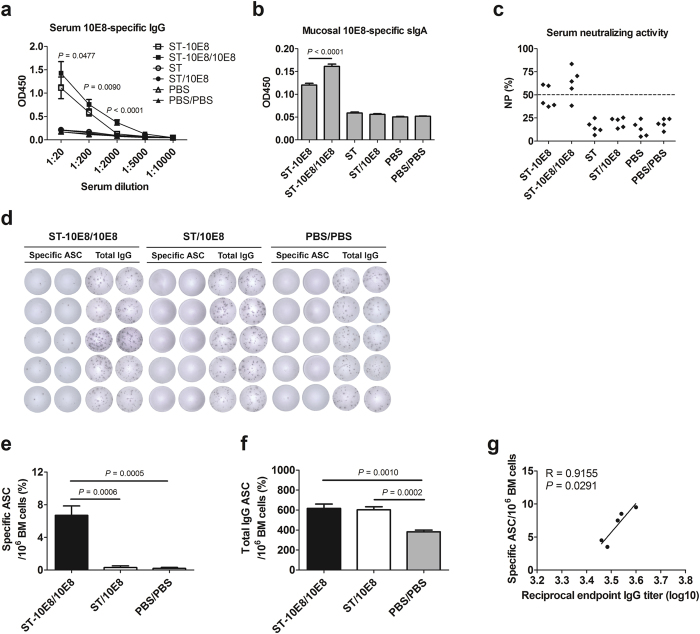
10E8 peptide specific antibody responses in serum and intestinal washes and antibody-secreting cells in bone marrow induced by oral immunization with ST-10E8. (**a**–**c**) Mice (5 per group) were orally primed with attenuated ST-10E8, ST or PBS, and boosted with 10E8 peptide for two times, and then 10E8 peptide specific serum IgG (**a**), intestinal wash sIgA (**b**) and serum anti-HIV-1 SF162 neutralizing antibody response (**c**) were detected. (**d**–**f**) Specific antibody secreting cells (ASCs) and total IgG ASCs at day 14 after the final boost were measured by B cell ELISpot assay. The spots of five mice in each group (**d**), the specific ASC (**e**) and total IgG ASC in 10^6^ bone marrow cells (**f**) were shown. (**g**) Correlation between specific antibody titer and the specific ASCs in bone marrow (BM) was shown. NP, neutralization percentage. R, correlation coefficient. Data shown as mean ± SD of 5 mice in each group.

BALB/c mice were vaccinated orally with the recombinant ST-10E8, empty vector ST or PBS for three times, and serum specific IgG and mucosal IgA titers were measured at day 14 after the final vaccination. Immunization with ST-10E8 induced a high level of serum 10E8-specific IgG response with an endpoint antibody titer of 1:1,859 (Fig. 2a). The 10E8 specific mucosal secretory IgA (sIgA) was also observed in the intestinal washes (Fig. 2b). Two of the five serum samples tested showed strong neutralizing activities against HIV-1 SF162 virus (NP value more than 50%) (Fig. 2c). No 10E8 peptide specific serum IgG, mucosal sIgA or neutralizing antibody response was detected in mice immunized with the ST vector or PBS.

Considering that the *Salmonella* vector itself contains multiple immunogens that might disturb the immunogenicity of heterologous antigens, we conducted additional homologous peptide boosting to drive the immune responses toward the 10E8 epitope more effectively and specifically. We performed two 10E8 peptide boosts and found that the ST-10E8 prime-10E8 boost regime gave approximate 2-fold increase (endpoint antibody titers of 1:3,273) of serum IgG titer than ST-10E8 immunization alone (Fig. 2a), significant improvement of mucosal sIgA responses in intestinal washes (Fig. 2b) and neutralizing antibody responses in four of five sera (Fig. 2c). Following the 10E8 peptide boosts, the ST primed mice (ST/10E8) did not produce significant 10E8 specific IgG, sIgA or neutralizing antibody response, consistent with previous findings that HIV-1 BNAb epitope peptide alone hardly induced effective specific antibody responses. These results suggest that ST-10E8 prime stimulated differentiation of specific T and B cells and promoted the formation of immune memory responses, contributing to rapid recall antibody responses following 10E8 peptide boosts.

### ST-10E8 stimulated differentiation of specific antibody-secreting plasma cells in bone marrow

We next measured the 10E8 epitope-specific antibody-secreting cells (ASCs) and total IgG ASCs in bone marrow after the final 10E8 peptide boost by B cell ELISpot assays. We found that ST-10E8/10E8 immunization induced significantly more specific ASCs and total IgG ASCs than PBS control group (Fig. 2d–f). The number of specific ASCs was positively correlated with the endpoint titer of specific serum IgG (Fig. 2g). Consistent with the results of specific antibody responses found in the above assays, ST/10E8 immunized mice did not generate obvious specific antibody-secreting plasma cells. Moreover, prime immunization with ST strains (ST-10E8 or ST) resulted in higher frequencies of total IgG ASCs than that with PBS, indicating that ST vector itself could facilitate B cell differentiation and would provide T hepler subsets or cytokine microenvironment helpful for specific antibody development.

### Attenuated ST immunization induced more serum IgG antibody and directed the immune responses towards Th1 bias

**Figure 3:**
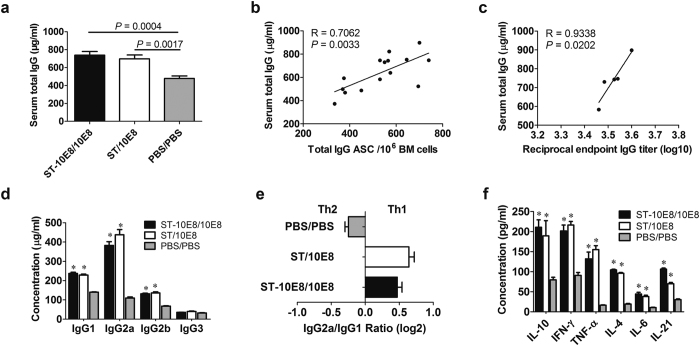
Total IgG, IgG subclasses and cytokine levels in sera of immunized animals. (**a**) Serum total IgG levels at day 14 after the final 10E8 peptide boost were measured by quantitative ELISA. The correlations between the serum total IgG concentration and total IgG ASC in BM (**b**), and the specific antibody titer (**c**) were shown. (**d**) IgG subclasses at day 14 after the final 10E8 peptide boost were analyzed by quantitative ELISA. (**e**) The Th1/Th2 bias of immunity was shown as the ratio of IgG2a to IgG1 in concentration. (**f**) The levels of Th1 cytokines (IL-10, IFN-γ, TNF-α) and Th2 cytokines (IL-4, IL-6, IL-21) in sera of immunized mice were detected by quantitative ELISA. R, correlation coefficient. Data shown as mean ± SD of 5 mice in each group. The asterisk indicates a significant difference versus PBS control (*P < 0.05).

Consistent with the results of total IgG ASCs in bone marrow, ST-10E8/10E8 and ST/10E8 immunized mice produced similar levels of serum total IgG antibodies, significantly higher than that in PBS control (Fig. 3a), suggesting that ST vector-specific IgGs were induced in the sera of the immunized mice. Correlation analyses showed that the serum total IgG concentration was positively associated with the total IgG ASC in bone marrow and the specific IgG antibody titer in serum (Fig. 3b,c). Both of the ST-10E8/10E8 and ST/10E8 groups also showed higher levels of serum IgG1, IgG2a and IgG2b subclasses than PBS group (Fig. 3d). No difference was observed in IgG3 levels among the three groups (Fig. 3d). Moreover, ST-10E8/10E8 and ST/10E8 immunization directed the immunity to Th1 bias as demonstrated by the ratio of IgG2a/IgG1 (Fig. 3e). Vaccination stimulation can change local cytokine microenvironment that will in turn influence the differentiation of T helpers, whose appropriate reaction is essential to the development and function of B cells. Therefore, we next assessed the cytokine profile in immunized mouse sera. We found that the sera of the ST-10E8/10E8 and ST/10E8 immunized mice contained significantly higher levels of both Th1 cytokines (IL-10, IFN-γ and TNF-α) and Th2 cytokines (IL-4, IL-6, and IL-21) than those of the PBS control mice (Fig. 3f), indicating that immunization with these STs induced mixed Th1 and Th2 immune responses and that cellular immunity and cytokine secretion induced by attenuated STs would further promote B cell differentiation and antibody production.

### Immunization with attenuated ST induced higher levels of splenocyte proliferation and B cell differentiation

**Figure 4:**
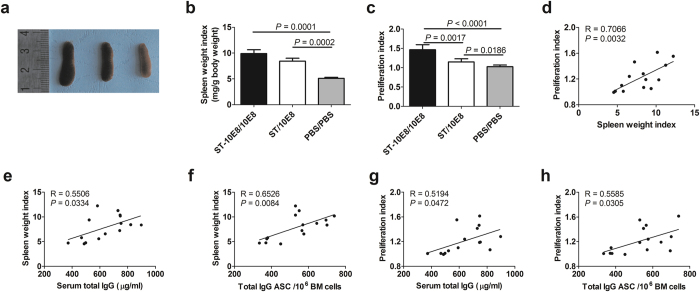
Spleen weight index and splenocyte proliferation activity. (**a**,**b**) The spleens from immunized mice were isolated and weighted, and spleen weight index were calculated as organ weight (milligram, mg) per gram (g) of mouse body weight. (**c**) The proliferation activity of splenic lymphocytes were measured under stimulation with 5 μg/ml 10E8 peptide or medium alone by CCK-8 assays. The proliferation index was expressed as the OD450 ratio of the peptide stimulation wells to the corresponding medium control wells. The correlations between the spleen weight index and the proliferation index (**d**), between the spleen weight index and serum total IgG (**e**), total IgG ASC (**f**), and between the proliferation index and serum total IgG (**g**), total IgG ASC (**h**) were shown. R, correlation coefficient. Data shown as mean ± SD of 5 mice in each group.

After the final boost, spleens and Peyer’s patches were isolated. As expected, we found that ST-10E8/10E8 and ST/10E8 immunized mice had much larger spleen weight indexes, as presented by the rate of spleen weight to mouse body weight, than PBS immunized mice (Fig. 4a,b), indicating that STs immunization induced significant proliferation of the immunized splenocytes. Then, we evaluated the 10E8 peptide specific proliferation under stimulation with the 10E8 peptide by CCK-8 assays, and found that the lymphocytes from ST-10E8/10E8 group showed significantly higher proliferative activity than that from ST/10E8 group, with proliferation indexes (PIs) of 1.46 and 1.15, respectively. The lymphocytes from PBS group showed no obvious proliferation activity against 10E8 peptide (PI = 1.02) (Fig. 4c). We also found that the spleen weight index was positively correlated with the splenocyte proliferation index (Fig. 4d), and both the spleen weight index and splenocyte proliferation index were positively correlated with the serum total IgG concentration and the total IgG ASC in bone marrow (Fig. 4e,f). These results suggested that immunization with recombinant *Salmonella* ST-10E8 stimulated proliferation and expansion of 10E8 specific lymphocytes as well as the formation of specific memory lymphocytes, which were rapidly activated by 10E8 peptide boosts, and that the attenuated ST vector induced non-10E8 specific splenocyte proliferation.

**Figure 5:**
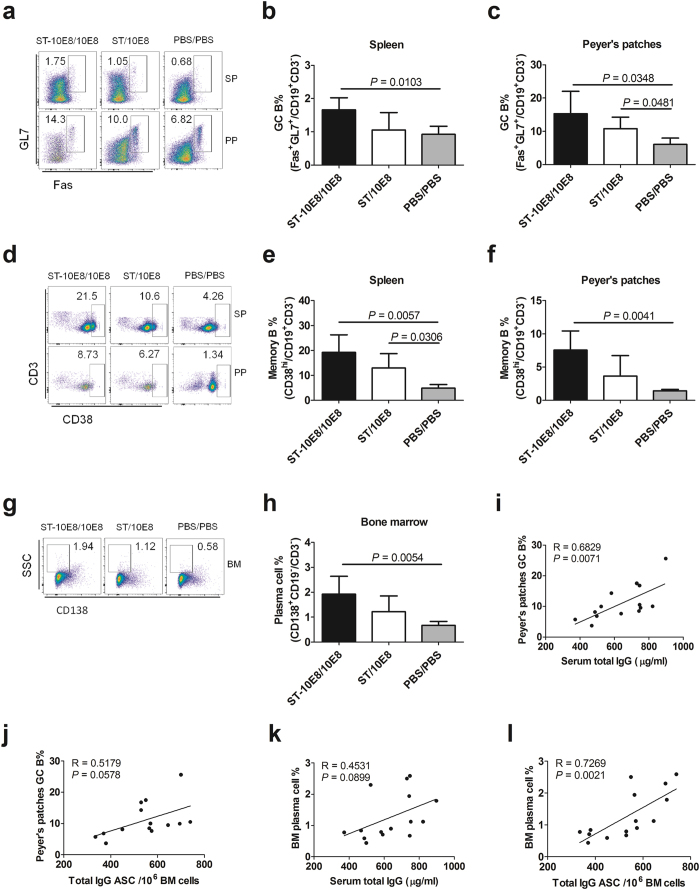
B cell subset differentiation induced by attenuated *Salmonella* immunization. (**a**–**h**) Contour plots and statistical graphs of GC B cells (Fas^+^GL-7^+^ CD19^+^CD3^−^) (**a**–**c**), memory B cells (CD38^hi^CD19^+^CD3^−^) (**d**–**f**) in spleen and Peyer’s patches, and plasma cells (CD138^+^CD19^−^CD3^−^) in bone marrow (**g**,**h**) at day 14 after final 10E8 peptide boost were shown. The correlations between the GC B frequency in Peyer’s patches and serum total IgG (**i**), total IgG ASC (**j**), and between plasma cell frequency in bone marrow (BM) and serum total IgG (**k**), total IgG ASC (**l**) were shown. R, correlation coefficient. Data shown as mean ± SD of 5 mice in each group.

We next wanted to know whether immunization with these STs could effectively induce B cell differentiation. Consistent with the above findings, mice immunized with ST-10E8/10E8 or ST/10E8 developed higher frequencies of GC B (Fas^+^GL-7^+^CD19^+^CD3^−^; Fig. 5a–c), memory B (CD38^hi^CD19^+^CD3^−^; Fig. 5d–f) in the spleen and Peyer’s patches, and plasma cells (CD138^+^CD19^−^CD3^−^; Fig. 5g,h) in bone marrow, than PBS immunized mice. The frequencies of these B subsets were slightly higher in ST-10E8/10E8 group than those in ST/10E8 groups (*P* > 0.05). The frequencies of the GC B cells in Peyer’s patches and the plasma cells in bone marrow were positively correlated with the serum total IgG concentration and the total IgG ASC in bone marrow (Fig. 5i–l). These results suggested that the attenuated STs prime could effectively stimulate germinal center formation and plasma cell development, and 10E8 boost might trigger 10E8 specific B cell differentiation.

## Discussion

It is well accepted that the elicitation of neutralizing Ab responses and induction of effective B cell responses play a central role in preventing HIV-1 infection^31^,^32^. Thus, an ideal vaccine candidate coupled with an appropriate vaccination regimen is needed desperately. However, various vaccine strategies for such a goal have all failed to date. In this study, we constructed a live attenuated *Salmonella*-vectored HIV-1 vaccine candidate that effectively expressed heterologous HIV-1 10E8 epitopes on bacterial fimbriae. We found that by using a recombinant bacterial ST-10E8 prime plus 10E8 peptide boost immunization regimen, this oral recombinant live bacterial vaccine could induce 10E8 epitope-specific antibody responses in peripheral and mucosal sites, and stimulate effective B cell differentiation.

HIV-1 Env is considered the primary target of neutralizing antibodies and comprises several well-defined regions that could be potential immunogens for vaccine development. The MPER of HIV-1 gp41 protein that is characterized as highly conserved among diverse HIV strains consists of several distinct short linear antigenic regions targeted by several BNAbs. However, when it comes to vaccine strategy, in order to generate effective Ab responses, the MPER antigens should be constructed and delivered in an appropriate way to improve the immunogenicity of the antigenic component^33^,^34^. Here, we employed *Salmonella* as the antigen delivery platform to present HIV-1 10E8 epitopes on its fimbriae. Our results showed that ST-10E8 could induce specific serum IgG and mucosal sIgA responses, and that *Salmonella* fimbriae display strategy could enhance the immunogenicity of the immunogens. Consistently, previous studies have reported that *Salmonella*-vectored DNA vaccine could significantly augment the systemic and mucosal antibody responses^23^,^28^,^29^. In addition, these findings are also consistent with our earlier work, in which we constructed *Salmonella* fimbriae based tumor vaccines and found that such a strategy can enhance the immunogenicity of tumor associated antigens and reduced the growth rate of the tumor cells^35^.

The “adjuvant effect” of *Salmonella* vectors in the enhancement of heterologous antigen immunogenicity seems to be derived from its capacity to modulate innate immune responses through the activation of TLR4 pathway^36^,^37^. Additionally, it has been reported that the flagellum TLR-mediated pathway is necessary for antibody responses^38^. But the mechanism for *Salmonella* fimbriae-mediated immunity enhancement is still unknown. Our results showed that immunization with attenuated *S. typhimurium* 14028-3b promoted production and secretion of several Th1 and Th2 type cytokines in serum, and stimulated B cell differentiation into GC B cells, memory B cells and plasma cells, indicating the immune activation effects of the *Salmonella* vector. Moreover, ST immunization seemed to direct the immune responses towards Th1 bias, consistent with previous findings^39^,^40^.

Granted that *Salmonella*-vectored immuogens came up with improved immunogenicity, the data in our experiments show that such improvement is not as profound as MPER vaccines in some previous reports^41^,^42^. Taken together, it indicates that immunological responses against the pathogenic bacterial vector certainly exist and that additional solutions are required to modulate the immune systems to target heterologous antigens specifically. In an ongoing study, we immunized mice with ST-10E8 up to five times with a three-week interval based on the bacterial clearance kinetics. The preliminary results showed that the serum 10E8 specific Ab reached the peak level at day 14 after 3rd immunization, and 4th and 5th immunizations did not yield any further improvement of specific Ab responses (data not shown). These findings suggest that more than three times of ST-10E8 immunization might lead to immune tolerance to the live *Salmonella* vector. Thus, in the present work, we determined to adopt 10E8 peptide boosts rather than repeat *Salmonella* immunizations. We found that boost vaccinations could enhance the Ab responses induced by ST-10E8 vaccination prime.

Moreover, further evaluation of plasma cell responses involving the plasma cell subsets in bone marrow and their antibody secreting function showed that the ST-10E8/10E8 regimen effectively drove B cells to focus on epitope-specific immune responses. Consistently, our analysis of the germinal center B cell responses occurring in spleens and Peyer’s patches supported these findings. The ST-10E8/10E8 vaccination regimen stimulated effective GC B cell responses, which is indispensable for later plasma B cell responses^43^,^44^.

To study the primary mechanisms underlying the B cell immune response stimulated by such vaccination strategy, we further evaluated serum cytokine profiles following last boost. We found that the ST-10E8/10E8 vaccination regimen stimulated mice to generate high levels of Th1 (IL-10, IFN-γ and TNF-α) cytokines and Th2 cytokines (IL-4, IL-6 and IL-21), with the Th1 cytokines being predominant. Additionally, the assessment revealed that the Tfh cytokine IL-21 was also effectively generated within the experimental group, which is consistent with the previous results regarding B cell responses following vaccination where IL-21 plays essential role in the formation and maintenance of germinal center activities^45^.

Overall, immunogenicity of the HIV-1 10E8 epitope was enhanced in our study by presenting the epitope peptide with the *Salmonella* fimbriae. The ST-10E8 prime and 10E8 epitope peptide boost vaccination regimen generated effective systemic and mucosal immune Ab responses to HIV-1 10E8 epitope. Furthermore, this regimen could drive more antibody secreting plasma cells specific to 10E8 epitope, which was supported by the analyses of germinal B cell responses and the assessment of serum cytokine profiles.

## Methods

### Bacterial strain and plasmid

The ST-14028-3b *aroA*^−^ strain (referred to as ST) was derived from *Salmonella typhimurium* 14028-3b and attenuated by deletion of AroA-coding gene. The plasmid pHSG415 contains a kanamycin-resistant gene for positive colony selection^46^ and was used in the construction of 10E8 epitope-expressing recombinant strain ST-10E8. The ST vector and the recombinant ST-10E8 strains were cultured in Luria Bertani (LB) medium at 37 °C.

### Construction of the recombinant ST-10E8 and AgfA deficient ST strains

The chimeric gene segment, *agfA::10E8*, was amplified by overlapping PCR as described previously^47^,^48^. Briefly, the upstream and downstream *agfA* segments containing the *10E8* insert were amplified by using primers agfA-for (5′-GCAGAATTCAGCAGTTGTAGTGCAGAAACAGTCGCATAT-3′)and 10E8-2 (5′-ACCGGATTTTATATACCACAGCCAGTTTGTTATGTCAAACCAATTGGATCCATTATCCGCACCCTGGCCTACATCG-3′), and the primers agfA-rev (5′-AGACGCAAGCTTCGTTTAATGTGACCTGAGGGATCACC-3′) and 10E8-1 (5′-GGATCCAATTGGTTTGACATAACAAACTGGCTGTGGTATATAAAATCCGGTGACCAGTGGAACGCTAAAAACTCCGAT-3′), respectively. Then, with the resulting gene segments as the PCR template, the chimeric gene segment, *agfA::10E8*, was amplified by using the primers agfA-for and agfA-rev. The final PCR product was inserted between the sites of *Eco*R I and *Hind* III of the plasmid pHSG415. After confirmation by sequencing, the recombinant plasmid pHSG415-agfA::10E8 was transformed into ST strain by electroporation, and the recombinant ST-10E8 strain was obtained by replacement of corresponding wild-type *agf*A gene segment in the chromosome with the chimeric *agfA::10E8* under selective pressure of ampicillin at 42 °C, as described previously^46^. The AgfA-deficient ST strain (ST-ΔAgfA) was constructed by the same procedure as described previously^49^.

### Western bolt analysis of fusion protein agfA-10E8

ST, ST-10E8 or ST-ΔAgfA were cultured on LB-agar plates, harvested and resuspended in Tris buffer (10 mM Tris, pH 7.5). The cell suspension was mixed with 6×  sample loading buffer (350 mM Tris-HCl pH 6.8, 3% glycerol, 10% SDS, 6% β-mercaptoethanol) and then boiled for 10 min. The insoluble components containing thin aggregative fimbriae AgfA were collected by centrifugation, and then treated with 90% formic acid for 45 s^49^. The formic acid was removed by Speed Vac Concentrator at 60 °C for 45 min^50^,^51^. The resultant protein samples were separated by SDS-PAGE, transferred to PVDF membrane, and detected using polyclonal rabbit anti-AgfA sera and monoclonal antibodies 10E8, 4E10, respectively. The mAbs 4E10 and 10E8 were obtained from the NIH AIDS Reagent Program, Division of AIDS, NIAID, NIH.

### Congo red binding assay

The amounts of Congo red (CR) bound by ST-10E8 and ST were determined by CR binding assay, as described previously^47^,^49^. Bacterial cells were cultured on LB agar plates supplemented with 100 μg/ml CR for 24 h. The cells were scrapped from the plate and resuspended to the A600 of 1 (1 × 10^9 ^CFU/ml) in 10 mM Tris-HCl (pH 7.0). The suspension was equilibrated for 1 h at room temperature (RT), and then 1 ml aliquots were transferred into tubes containing 50 μl 30% PEG8000. The bacterial cells were removed from the tube by centrifugation at 15,000 × *g* for 5 min. The amounts of CR released into the supernatants were measured by reading optical density (OD) value at 480 nm.

### Mice, oral immunization and sample collection

Female BALB/c mice (8–10-week old) were purchased from Vital River Laboratories (Beijing, China) and housed in the Animal Laboratory of Harbin Medical University. The methods of animal experiments were carried out in strict accordance with the recommendations in the Guide for the Care and Use of Laboratory Animals of the National Institutes of Health. All experimental protocols were approved by Animal Care and Use Committee of Harbin Medical University (HMUIRB20150024).

In order to measure the LD50 of ST stains, 10 mice per group were orally administrated with ST-10E8 or ST ranging from 1 × 10^7^ to 1 × 10^11^ CFU. The survival rate of infected mice was monitored weekly for a 28-day period. In order to examine the organ distribution of ST strains, 12 mice per group were infected orally with 1 × 10^8^ CFU of ST-10E8 or ST. At days 3, 9, 16, 30 post infection, the spleens and Payer’s patches were isolated to determine the bacterial loads by plating the organ homogenates on LB-agar plates as previously described^52^,^53^.

For immunogenicity evaluation, 5 mice per group were inoculated orally with 1 × 10^8^ CFU of ST-10E8, ST or PBS at weeks 0, 3 and 6. Blood samples were collected via the tail vein at day 14 after the third vaccination. All ST-10E8 or ST primed mice were boosted intraperitoneally with 20 μg 10E8 peptide emulsified in Freund’s adjuvant at weeks 9 and 12. The PBS-primed mice were boosted with the adjuvant alone. The sera, spleens, Payer’s patches and bone marrows were collected at day 14 after the second 10E8 peptide boost. A 10-cm intestinal segment in duodenum region from each mouse was also collected, cut into 1-cm length pieces and washed thoroughly with 1 ml PBS. The intestinal washes were used for mucosal IgA detection.

### Measurement of 10E8 peptide specific binding antibody responses

10E8 epitope-specific binding antibodies in serum and intestinal washes were analyzed by Enzyme-linked immunosorbent assay (ELISA). Briefly, 96-well plates were coated with purified synthetic 10E8 epitope peptide (NWFDITNWLWYIK) at the final concentration of 0.5 μg/ml, and incubated at 4 °C for 12 h. The plates were washed three times with PBS containing 0.05% Tween-20 (PBST) and then were blocked with the blocking buffer (2% skim milk in PBST) at 37 °C for 1 h. After washings, intestinal washes or serially diluted mouse sera were added and incubated at 37 °C for 1 h. After washings, horseradish peroxidase (HRP)-conjugated goat anti-mouse IgA or IgG (ZSGB-BIO, Beijing, China) was added and incubated at 37 °C for 1 h. And then, 3,3′,5,5′-tetramethy1 benzidine (TMB) substrate was added and incubated at RT for 30 min. The reaction was stopped by addition of 50 μl of 2 M H_2_SO_4_ solution. The absorbance value at 450 nm (OD450) was read using ELISA reader (Bio-Rad, California, USA).

### Detection of anti-HIV-1 neutralizing activity of sera from immunized mice

Neutralization assays on HIV-1 SF162 Env pseudovirus were performed in TZM-bl cells as previously described^54^. Briefly, mouse sera were heat-inactivated at 56 °C for 1 h prior to the assay, and 1:10 diluted in DMEM containing 10% heat-inactivated fetal bovine serum. A one hundred 50% tissue culture infective dose (TCID50) of SF162 Env pseudovirus (25 μl) and the diluted serum (25 μl) were mixed, added to a 96-well plate in duplicate and incubated at 37 °C for 1 h. Then, 50 μl TZM-bl cell suspension containing 1 × 10^4^ cells was added to each well. After incubation at 37 °C for 60 h, 100 μl of Bright-Glo Luciferase assay System (Promega, Wisconsin, USA) was added to each well and incubated at RT for 5 min. The relative luminescence unit (RLU) in each well was read with a ModulusII microplate multimode reader (Turner BioSystems, California, USA). The neutralization activity of the mouse sera was expressed as neutralization percentage (NP) calculated as the percentage of reduction in RLU values in wells containing pseudovirus and serum relative to the RLU values in wells containing pseudovirus alone. The TZM-bl cell line was obtained from the NIH AIDS Reagent Program, Division of AIDS, NIAID, NIH.

### Analysis of antibody secreting cells in bone marrow

The 10E8 peptide specific and total IgG antibody secreting cells (ASCs) were detected by B-ELISPOT assays using the ELISpot^PLUS^ for Mouse IgG kit (Mabtech, Nacka Strand, Sweden) as described previously^55^. Briefly, the 96-well plate was pre-wetted with ethanol, and then added with anti-mouse IgG antibody (15 μg/ml) and incubated overnight at 4 °C. After washes with PBS, the plate was blocked with RPMI1640 medium containing 10% fetal calf serum (FCS) for 30 min at RT. After removal of blocking buffer, 1 × 10^5^ or 1 × 10^6^ bone marrow cells per well, for total IgG ASCs or for specific IgG ASCs detection, respectively, were added and incubated at 37 °C, 5% CO_2_ for 22 h. After removal of the cells and washes, the plates were further incubated with anti-IgG-biotin or biotin-labeled 10E8 peptide. Following additional incubation with Streptavidin-ALP (1:1,000) at RT for 1 h, the plates were developed with BCIP/NBT solution. The numbers of the cell spots were counted using an ELISPOT reader (Sage Creation Science, Beijing, China).

### Analysis of serum IgG subclasses and the Th bias of immunity induced by attenuated STs

Serum total IgG was quantitatively measured by using the Mouse IgG total ELISA Ready-SET-Go Kit (eBioscience, San Diego, California, USA) following the manufacturer’s instructions. Briefly, the anti-IgG antibodies were added onto 96-well plates and incubated overnight at 4 °C. After washing with PBST and blocking, the serum samples (1: 10,000) or 2-fold serially diluted IgG standards, and the detection antibodies were incubated at RT for 3 h. Then, the substrate solutions were added and incubated at RT for 15 min. The stopping of reactions and the reading of OD450 values were performed as described above.

IgG subclass concentrations in mouse sera were quantitatively detected by using Mouse IgG Subclass ELISA kits (Enzyme-linked Biotechnology Co., Ltd., Shanghai, China). Briefly, serum samples from immunized mice were diluted and added into the 96-well plates that were pre-coated with anti-mouse IgG1, IgG2a, IgG2b or IgG3 antibodies, and then incubated at 37 °C for 30 min. After five times of washes, HRP-conjugated anti-mouse IgG1, IgG2a, IgG2b or IgG3 antibodies were added. After incubation and washes, the substrate solution was added into each well for color development. The stopping of reactions and the reading of OD450 values were performed as described above. The concentrations of IgG subclasses in mouse sera were calculated according to the standard curves, which were constructed by using the standard mouse IgG1, IgG2a, IgG2b or IgG3 proteins.

### Measurement of serum cytokine profiles

Secretory levels of cytokines IL-4, IL-6, IL-10, IL-21, IFN-γ and TNF-α in the mouse sera were quantitatively determined using the ELISA kits (BOSTER, Wuhan, China) according to the manufacturer’s instructions. Briefly, sera from the immunized mice (1:10 dilution) were added to the cytokine-specific antibody coated 96-well plates and incubated at 37 °C for 1.5 h. After removal of unbound serum proteins, biotin-labeled cytokine-specific detection antibody was added and incubated at 37 °C for 1 h. The plates were washed three times and then were incubated with streptavidin-HRP for 30 min. After washes, the TMB substrate solution was added, and color reaction was developed. The stopping of reactions and the reading of OD450 values were performed as described above. Cytokine concentrations were calculated by using the standard curves derived from the recombinant cytokine standards.

### Antigen-specific splenocyte proliferation assay

Splenocytes from immunized mice were harvested and lymphocytes were isolated. The lymphocytes (5 × 10^5 ^cells/well) were incubated with 5 μg/ml of 10E8 or medium alone (unstimulated) in duplicate for 72 h at 37 °C. The proliferation activity was examined by Cell Counting Kit-8 (Dojindo Molecular Technologies, Tokyo, Japan). The proliferation index was calculated as the ratio of OD450 values in stimulated cells to those in the corresponding unstimulated control.

### Flow cytometry analyses

Suspensions of single lymphocytes from spleens and Payer’s patches were prepared and stained with antibodies as follows: anti-CD3-pacific blue, anti-CD19-PE/Cy7, anti-CD38-APC, anti-CD138-biotin, anti-Fas-PE/CF594, anti-GL7-FITC and Streptavidin-PE from BD Biosciences (California, USA). The lymphocytes from bone marrows were isolated and stained with anti-CD3-pacific blue, anti-CD19-PE/Cy7, and anti-CD138-biotin. Flow cytometry data were acquired by the flow cytometer (CantoII; BD Biosciences) and analyzed with FlowJo software (version 10; Tree Star Inc, Oregon, USA).

## Statistics

Statistical analyses were performed by using GraphPad Prism version 5.0 (GraphPad Software, California, USA). The data were expressed as mean ± SD. A students’ *t*-test (two-tailed) was used to compare the means between any two groups. Pearson’s correlation coefficient was employed to determine linear relations between two parameters. *P* values less than 0.05 were considered statistically significant.

## Additional Information

**How to cite this article**: Li, Q.-H. *et al.* Live attenuated *Salmonella* displaying HIV-1 10E8 epitope on fimbriae: systemic and mucosal immune responses in BALB/c mice by mucosal administration. *Sci. Rep.* **6**, 29556; doi: 10.1038/srep29556 (2016).
